# Ceruminous adenoma of the external auditory canal: 9 cases series with imaging and pathologic findings

**DOI:** 10.3389/fonc.2023.1041282

**Published:** 2023-07-06

**Authors:** Yifan Yang, Honggang Liu, Jugao Fang, Yongxin Li, Shubin Chen

**Affiliations:** ^1^ Department of Otorhinolaryngology Head and Neck Surgery, Beijing Tongren Hospital, Capital Medical University, Beijing, China; ^2^ Department of Pathology, Beijing Tongren Hospital, Capital Medical University, Beijing, China

**Keywords:** external auditory canal, ceruminous glands, adenoma, clinical feature, pathological feature

## Abstract

**Objectives:**

Ceruminous adenoma is a rare benign tumor of the external auditory canal. This study aimed to present the clinical characteristics, imaging findings, pathological results and the management outcomes of the ceruminous adenoma.

**Study design:**

Retrospective case series review.

**Setting:**

Tertiary referral center.

**Patients and methods:**

Patients undergoing surgery for ceruminous adenoma of the external auditory canal between the years 2004 to 2018. All patients with ceruminous adenoma were analyzed for demographic, clinical, radiological features and pathologic findings. The outcomes of the management were also evaluated.

**Results:**

Nine patients with ceruminous adenoma were included in the study. Hearing loss was the most common complaint (5/9, 56%), followed by otalgia (4/9, 44%), pruritus (4/9, 44%), and otorrhea (2/9, 22%). The tumors originated mostly from the cartilaginous portion of the external auditory canal (8/9, 89%) and merely from the bony portion of the external auditory canal (1/9, 11%). Pathohistological study indicated that the ceruminous adenomas were divided into three types: the ceruminous gland adenoma (6/9, 67%), the ceruminous pleomorphic adenoma (2/9, 22%) and the ceruminous syringocystadenoma papilliferum (1/9, 11%). No recurrence was found during follow-up for two to fifteen years after surgical resection.

**Conclusion:**

Ceruminous adenomas are rare entities. They originate mainly from the cartilaginous portion of the EAC, but occasionally from the bony portion of the EAC. The surgical section with enough margin is adequate for management of these tumors.

## Introduction

Ceruminous glands are modified apocrine glands located primarily in the outer one-third to one-half of the external auditory canal (EAC). Ceruminous gland tumors of the EAC are very rare clinical–pathological entities (1). The benign tumor of the ceruminous glands is also referred to as ‘ceruminous adenoma (CA)’, which shows a dual population of a basal, myoepithelial layer surrounding inner luminal secretory cell (2, 3) and is usually separated into three types, based on histologic features: ceruminous gland adenoma, ceruminous pleomorphic adenoma, and ceruminous syringocystadenoma papilliferum (1, 4, 5). CA has a good prognosis after complete excision. The literature on these tumors is limited to case reports and small group cases, and there is a lack of comprehensive evaluation. We present herein 9 cases of ceruminous adenomas at a tertiary care center and briefly describe the salient clinical data, imaging findings, pathological features, treatment modalities, to raise awareness of this rare entity.

## Methods

Following Institutional Review Board approval from Beijing Tongren hospital, a retrospective chart review was performed to identify all patients with histologically confirmed ceruminous adenoma between 2004 and 2018. Nine patients diagnosed with ceruminous adenomas of the EAC were found. The following data were extracted: patient demographics, presentation, imaging, histopathology, treatment, follow-up, recurrence, and final outcome of disease.

## Results

### Demographics, symptoms, and signs

There were a total of five males and four females with a diagnosis of ceruminous adenoma of the EAC. The mean age at presentation was 51 years old, with a range from 23 to 84 years old. The most common presenting symptom was hearing loss (5/9, 56%), followed by otalgia (4/9, 44%), pruritus (4/9, 44%), and otorrhea (2/9, 22%). Tinnitus was less common (1/9, 11%). Duration of symptoms before admission ranges from one month to 17 years. Clinical findings included a mass that was present in the ear (9/9, 100%), followed by purulent external auditory canal exudate (2/9, 22%). (see **Table 1**).

**Table 1:** Clinical demographics, symptoms, and signs.

Case No	Age/sex	Symptoms	Duration	Site in the EAC	Size (mm)	Margin	Gross appearance	Histolog- ic type
1	23/M	Hearing loss Otorrhea	1 year	CP Extending to BP	25	Clear	Ovale Solid	CGA
2	46/F	Hearing loss Ear pain Tinnitus	1 month	BP	10	Clear	Cystic-solid	CGA
3	41/F	Pruritus	1 year	CP	11	Clear	Ovale solid	CPA
4	59/M	Hearing loss Pruritus	3 years	CP	12	Clear	Round solid	CGA
5	71/M	Hearing loss	3 years	CP	16	Clear	Ovale lobulated solid	CGA
6	44/F	Pruritus	10 months	CP	10	Clear	Cystic-solid	CGA
7	33/F	Ear pain	1 years	CP	4	Clear	Round Solid	CGA
8	84/M	Hearing loss Ear pain Otorrhea	3 years	CP extending out of the EAC and to the parotid	36	Clear	lobulated Solid	CPA
9	61/F	Hearing loss Ear pain Pruritus	17 years	CP	17	Clear	Cystic-solid	CSCAP

CP, cartilaginous portion of the EAC; BP, bony portion of the EAC; CGA, Ceruminous gland adenoma; CPA, Ceruminous pleomorphic adenoma; CSCAP, Ceruminous syringocystadenoma papilliferum.

### Radiographic findings

**Figure 1:**
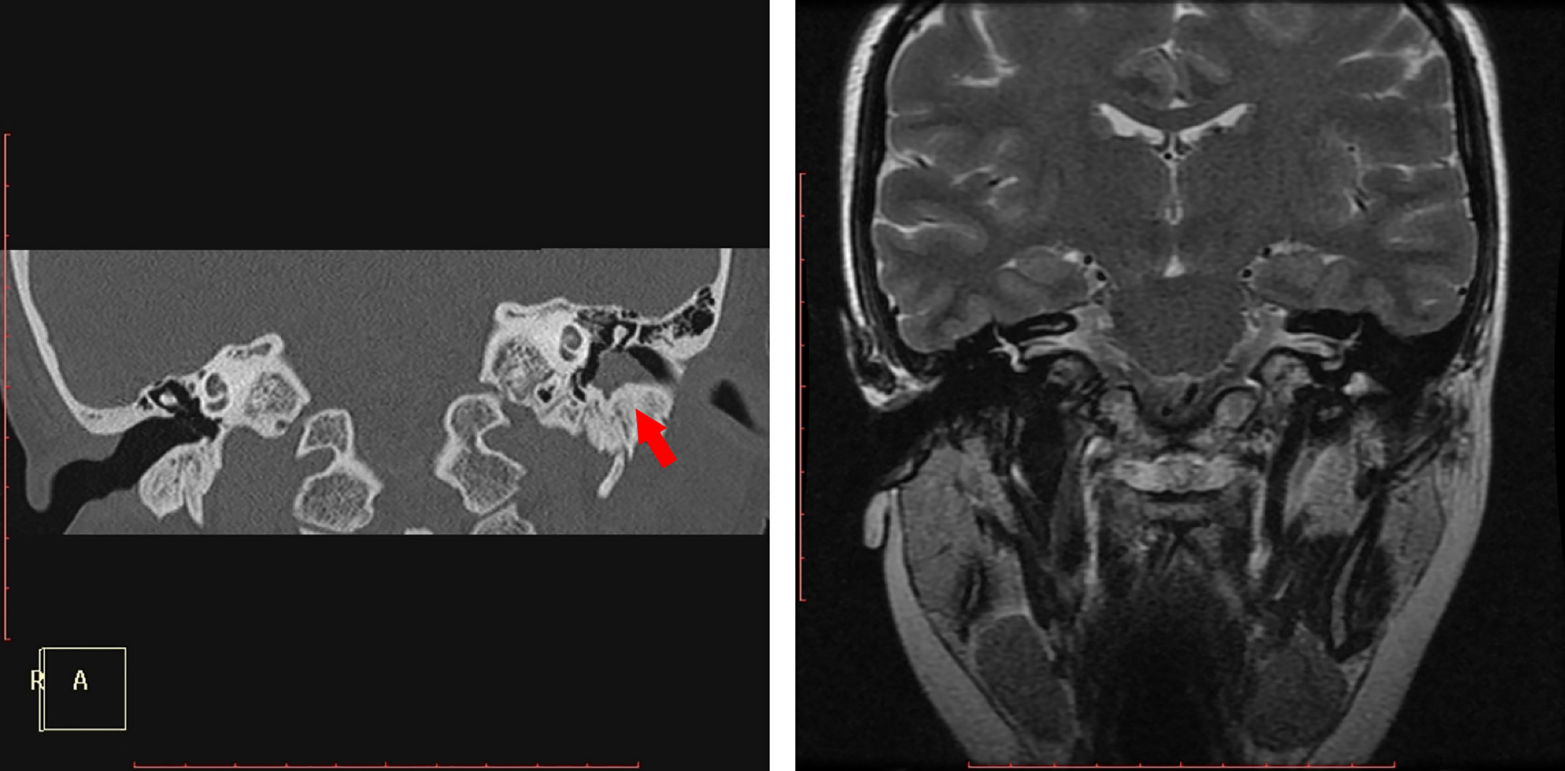
(case 2): Ceruminous gland adenoma in the bony portion of the left external auditory canal. Coronal CT showed a soft mass, the tympanic bone was mildly involved and was highlighted by a red arrow(a). Coronal T2WI showed a high signal intensity in the deep part of the EAC.

High-resolution computerized tomography (HRCT) was obtained for 8 of 9 patients and magnetic resonance imaging (MRI) was acquired for 7 of 9 of the patients. All patients demonstrated a soft mass in the EAC. The masses ranged in size from 4 to 36 millimeter in greatest dimension (mean, 17 mm). The site of the tumors is summarized as follows: in six cases (6/9, 67%) the lesions were located only in the cartilaginous portion of the external auditory canal (EAC); in one case (1/9, 11%) the lesion was located only in the bony portion of the external auditory canal (EAC) (**Figure 1**); in one case (1/9, 11%) the lesion was originated from the cartilaginous portion with extension into the bony part of the EAC; in another case (1/9, 11%), the tumor located in the cartilaginous portion and protruded out from the EAC. The tumor margin was well circumcised and confined in all cases.

**Figure 2:**
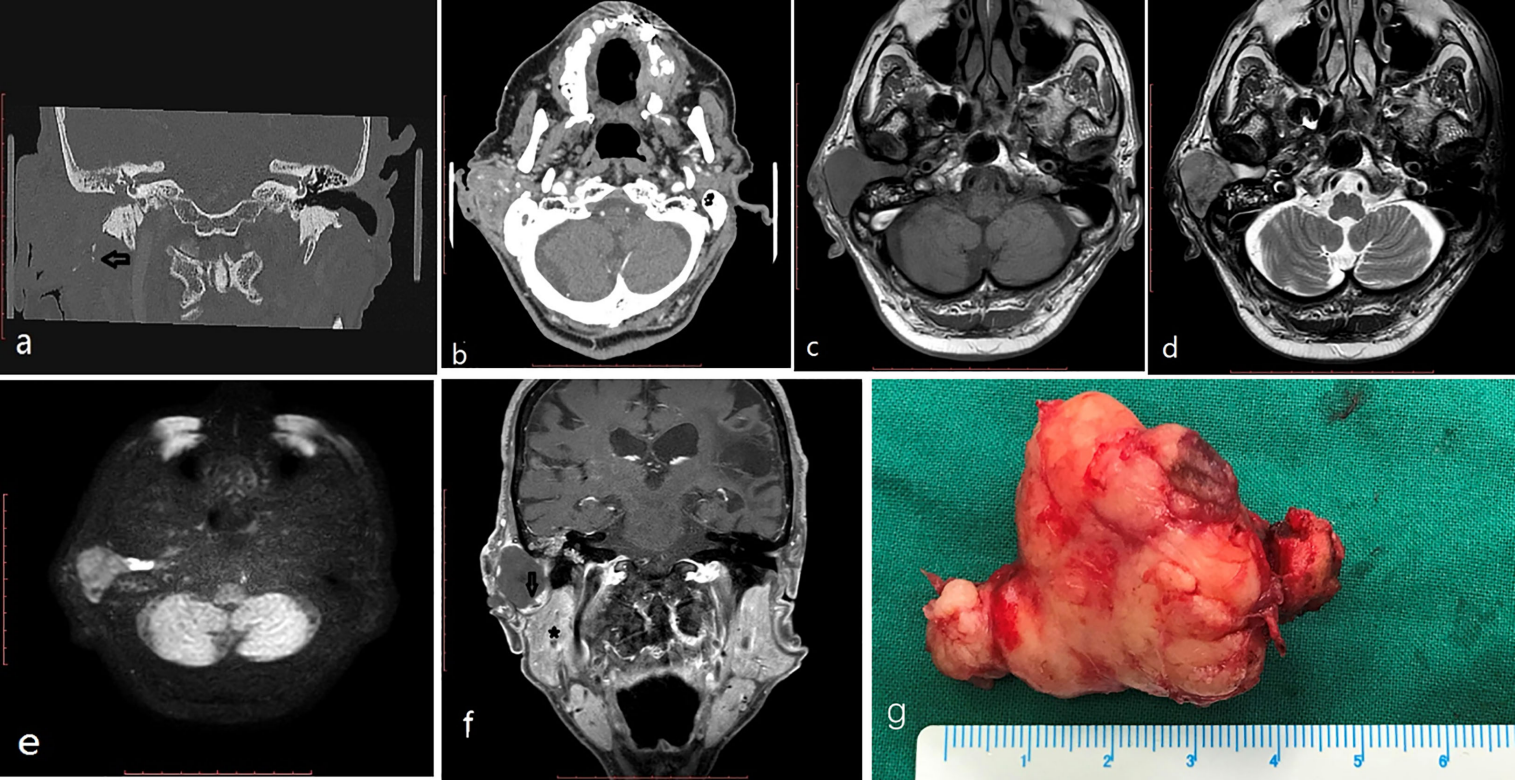
(case 8) An 84-year-old man had a 36mm max diameter ceruminous pleomorphic adenoma in the right EAC protruding out from the EAC and extending to the parotid (behind the mass, cholesteatoma was found in the EAC). CT: **(A, B)** calcified foci (black arrow) and heterogenous enhancement in the tumor. MRI **(C-F)**:T1 low intensity **(C)**, T2 intermediate and high signal intensity **(D)**, DWI intermediate signal intensity **(E)** (behind the mass, cholesteatoma appeared as high intensity); the mass showed edge enhancement (black arrow) and was adjacent to the parotid (star) on coronal enhanced T1 **(F)**. The mass is a lobulated solid mass on the gross appearance **(G)**.

On temporal HRCT, all of the lesions exhibited isoattenuating soft tissue masses in the EAC, one mildly involved the tympanic bone (**Figure 1**). In one patient several calcification foci were found in the mass, and the mass showed heterogenous enhancement on the CT with the soft window. Seven cases were examined with MRI, six cases underwent contrast-enhanced MRI, and DWI was performed in five cases. On T1WI, four cases demonstrated intermediate signal intensity, two cases showed mixture of intermediate and high signal intensity, and one case showed low signal intensity. On T2WI, four cases demonstrated high signal intensity, two cases showed mixture of intermediate and high signal intensity, and one case showed intermediate signal intensity. On contrast-enhancement T1WI, three cases showed edge enhancement, two cases homogenous enhancement, one case heterogenous enhancement. On DWI of five cases, all tumors appeared as intermediate signal intensity, but in two of them high signal intensity of cholesteatoma was also found deep to the tumor in the EAC (**Figures 2D, F**). **Figure 2** showed that the tumor was adjacent to the parotid gland, however, the **Figure 2F** revealed the tumor was originated in the EAC, there was a gap between the tumor and the parotid gland, and **Figure 2G** revealed the integrity of the tumor capsule. Thus, the tumor did not contain any salivary gland tissue, and the tumor originated in the EAC (See **Table 2**).

**Table 2:** Summary of CT and signal intensity on MRI.

Case no	CT	SI on MRI			
		T1W1	T 2W1	Contrast enhancement	DWI
1	Soft mass	N/A	N/A	N/A	N/A
2	Bone involvement	Intermediate and high	High	Edge enhancement	N/A
3.	Soft mass	N/A	N/A	N/A	N/A
4	Soft mass	Intermediate and high	Intermediate	**Homogenous enhancement**	N/A
5	Soft mass	Intermediate	High	N/A	Intermediate
6	Soft mass	Intermediate	Intermediate and high	Heterogenous enhancement	Intermediate
7	Soft mass	Intermediate	High	**Homogenous enhancement**	Intermediate
8	Soft mass with calcified foucus and heterogenous enhancement.	Low	Intermediate and high	Edge enhancement	Intermediate
9	N/A	Intermediate	High	Edge enhancement	Intermediate

SI, Signal intensity.

N/A, not available.

### Histology

**Figure 3:**
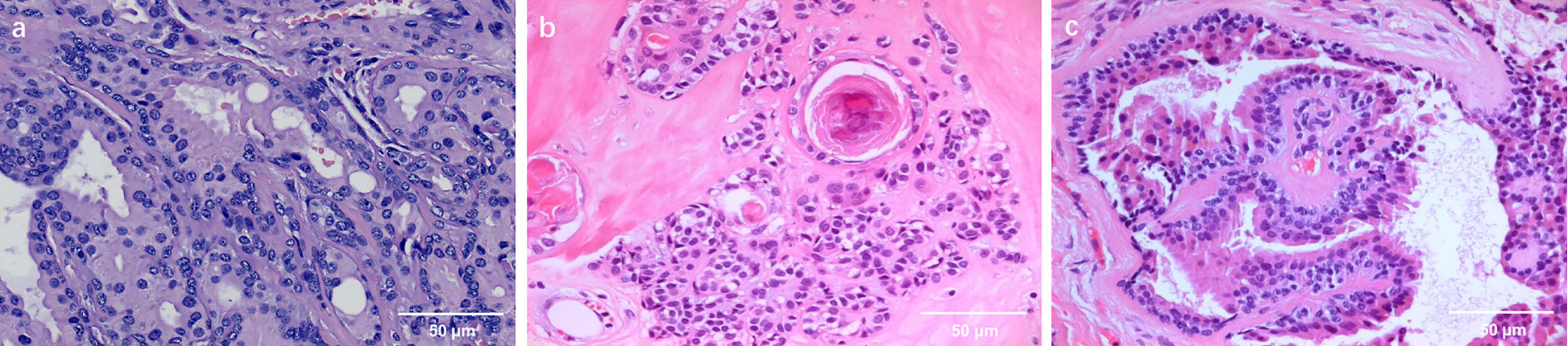
**(A)** (case 5) Ceruminous gland adenoma of the external auditory canal, evaluation with hematoxylin-eosin staining. The neoplastic proliferation of ceruminous glands is visible. The glandular epithelium is bilayered. Abundant cytoplasm is seen in the luminal cells, which show focal decapitation secretion and contain ceroid pigment (40x). **(B)** (case 8) Histological findings of ceruminous pleomorphic adenoma of the EAC showing the glandular epithelial component and myxofirous stroma(40x). **(C)** (case 9) Ceruminous syringocystadenoma papilliferum of the external auditory canal, evaluation with hematoxylin-eosin staining. Note papillae lined by glandular epithelium projecting into a cystic lumen(40x).

On gross appearance, 6 lesions appeared as solid masses, 3 cases appeared as cystic-solid masses; the masses were round, ovale, or lobulated. According to the 2017 WHO classification of ceruminous neoplasms, nine cases were diagnosed as benign ceruminous adenoma. Among them, six cases were diagnosed as ceruminous gland adenoma (**Figure 3A**), two cases were diagnosed as ceruminous pleomorphic adenoma (**Figure 3B**), one case was diagnosed as ceruminous syringocystadenoma papilliferum (**Figure 3C**).

### Treatment

Seven patients were operated under general anesthesia and two patients were operated under local anesthesia. Six patients underwent endaural microscope surgery, two patients underwent transcanal endoscope surgery, and one patient underwent postauricular approach surgery. Postaural free skin flaps were used in seven patients. No recurrence was found in all patients during the follow-up from 3 to 17 years.

## Discussion

Ceruminous glands are modified apocrine glands located mainly in the skin lining cartilaginous portion of the external auditory canal (EAC) (2). The medial bony portion of the EAC is normally free from these glands, although they can be seen rarely as an incidental finding.

Tumors of ceruminous gland are extremely uncommon in humans. Currently, World Health Organization (WHO) recognizes 2 categories of ceruminous neoplasms: benign (ceruminous adenoma) and malignant (ceruminous adenocarcinoma) (3, 5). According to histology ceruminous adenoma included three most common entities: ceruminous gland adenoma, ceruminous pleomorphic adenoma and ceruminous syringocystadenoma papilliferum. Ceruminous adenocarcinoma included adenoid cystic carcinoma; adenocarcinoma, mucoepidermoid carcinoma.

Ceruminous adenomas develop mainly in the cartilaginous portion of the external auditory canal, rarely in the osseous portion (6, 7). In our series, eight cases of ceruminous adenoma originated from cartilaginous portion of the EAC, only one case originated from bony portion of it (**Figure 1**).

The typical symptoms of ceruminous adenoma, such as conductive hearing loss, otorrhoea and pain, are usually related to the size of the lesion and the degree of canal obstruction. It’s also entirely possible that squamous epithelial keratin debris could accumulate behind the mass leading to the problem of cholesteatoma. The benign adenoma may exist for many years. The longest disease duration before admission in our series is seventeen years. Ceruminous adenoma is typically a skin- covered, round or ovale, polypoid or lobulated mass of variable size in the EAC. It may demonstrate a cystic to solid pattern. In our series, 6 cases present as solid mass, 3 cases present as cystic-solid mass.

*CT* as *a* routine *examination can* determine the exact location of the tumor and evaluate whether there is bone destruction or not. On CT examination ceruminous adenomas usually present as a soft mass. In our series there was no tympanic bone destruction in all cases except one case, in which only mild bone destruction was found. Calcification foci were found in one of our cases with ceruminous pleomorphic adenoma (**Figures 2A, B**). Calcification in ceruminous adenoma was not reported in the literature. The calcification is thought to be a result of long-term tumor progression in pleomorphic adenoma of other sites. The frequency of calcification was 50% in parapharyngeal space pleomorphic adenoma and 15% in parotid pleomorphic adenoma (8, 9).

MRI can determine the extent and margin of the tumor. Ceruminous adenoma usually demonstrated a well-circumicised mass without infiltration to adjacent tissue. In one of our cases the tumor growed adjacent to the parotid but showed no infiltration. They might show intermediate, high or low signal intensity on T1WI, and show intermediate or high signal intensity on T2WI. They may show homogenous, or heterogenous, or edge enhancement. On DWI they showed as intermediate signal, but cholesteatoma behind the tumor appeared as high signal (**Figure 2E**).

The benign tumors of ceruminous gland are separated into three histologic types: ceruminous adenoma, ceruminous pleomorphic adenoma, ceruminous syringocystadenoma papilliferum. All these tumors are unencapsulated, but are usually well circumscribed or defined (5). The overlying surface epithelium is usually not involved. They showed no infiltration to other tissue.

Ceruminous gland adenomas (CGA) are the most common benign glandular tumors of the EAC. The lesion consists of glandular structures lined by two-layers of epithelium typically arranged in lobulated clusters (**Figure 3A**). The inner luminal secretory cells display abundant eosinophilic cytoplasm with scattered yellowish-brown ceroid cytoplasmic pigment granules, while the myoepithelial cells line up along the basement membrane. Cystic and solid pattern of growth may be present.

Ceruminous pleomorphic adenoma (CPA), also referred to as mixed tumor, is the second most common benign glandular neoplasm of the EAC. It is characterized by a mixed cellular presence consisting of the epithelial cells lining the lumens of tubular formations surrounded by nests of myoepithelial cells within a myxochondroid or even mucoid matrix (**Figure 3B**) (10, 11). Myoepithelial cells of the ceruminous glands are thought to be the precursor of primary pleomorphic adenoma of the ear canal. Ceruminous pleomorphic adenoma is identical to salivary gland pleomorphic adenoma, requiring careful exclusion of extension from a primary parotid gland tumor into the EAC.

Ceruminous syringocystadenoma papilliferum is an extremely rare lesion in the EAC, only a dozen of cases were reported (12–16). It specifically contains papillae lined by two-layered epithelium projecting into cystic lumens (**Figure 3C**). Luminal cells usually reveal prominent apical caps with decapitation secretion, typical of the ceruminous glands. It contains solid and cystic areas.

The mainstay in the management for all benign ceruminous gland tumors of the external ear canal is en bloc surgical resection with a negative margin (5). Transmeatal excision is usually adequate for small tumors, while postauricular approach may be needed for larger tumors. A free skin flap is usually recommended for reconstruction. The possibility of recurrence for these benign tumors seems to be related only to an incomplete surgical excision of the lesion (17). Although the recurrence rate is low, long-term follow-up is needed. Radiation and chemotherapy are not used to treat these benign neoplasms.

## Conclusion

Ceruminous adenomas are benign tumors of the EAC. They are rare entities. They originate mainly from the cartilaginous portion of the EAC, but occasionally from the bony portion of the EAC. The ceruminous gland adenoma is the most common histologic type of benign glandular neoplasms, the ceruminous pleomorphic adenoma is the second most common of these neoplasms, and the ceruminous syringocystadenoma papilliferum is an extremely lesion in the EAC.Calcification can be found in the large ceruminous pleomorphic adenoma. The surgical section with enough margin is adequate for management of these tumors.

## Data availability statement

The original contributions presented in the study are included in the article/supplementary material. Further inquiries can be directed to the corresponding author.

## Ethics statement

The studies involving human participants were reviewed and approved by Ethics Committee of Beijing Tongren Hospital, Capital Medical University. The patients/participants provided their written informed consent to participate in this study. Written informed consent was obtained from the individual(s) for the publication of any potentially identifiable images or data included in this article.

## Author contributions

Conceptualization: SC. Data curation: HL, YL. Methodology: HL, SC. Project administration and Supervision: SC. Validation: JF. Writing: YY, HL. All authors contributed to the article and approved the submitted version.
